# Prevalence of Sleep Disordered Breathing in Patients with Primary Mitral Regurgitation Undergoing Mitral Valve Surgery

**DOI:** 10.3390/jcm10092039

**Published:** 2021-05-10

**Authors:** Muhammed Gerçek, Olaf Oldenburg, Mustafa Gerçek, Henrik Fox, Volker Rudolph, Thomas Puehler, Hazem Omran, Lisa Katharina Wolf, Kavous Hakim-Meibodi, Andreas M. Zeiher, Jan Gummert, Zisis Dimitriadis

**Affiliations:** 1 Clinic for General and Interventional Cardiology/Angiology, Herz-und Diabeteszentrum NRW, Ruhr-Universität Bochum, 32545 Bad Oeynhausen, Germany; mugercek@hdz-nrw.de (M.G.); hfox@hdz-nrw.de (H.F.); vrudolph@hdz-nrw.de (V.R.); homran@hdz-nrw.de (H.O.); l.k.wolf88@gmail.com (L.K.W.); 2 Clinic for Cardiology, Ludgerus-Kliniken Münster, 48153 Münster, Germany; o.oldenburg@alexianer.de; 3 Clinic for Cardiovascular Surgery, Heart Center Duisburg, 47137 Duisburg, Germany; Mustafa.Gercek@evkln.de; 4 Department of Cardiac and Vascular Surgery, Campus Kiel, University Medical Center Schleswig Holstein, 24105 Kiel, Germany; Thomas.Puehler@uksh.de; 5 Clinic for Thoracic and Cardiovascular Surgery, Herz-und Diabeteszentrum NRW, Ruhr-Universität Bochum, 32545 Bad Oeynhausen, Germany; khakim-meibodi@hdz-nrw.de (K.H.-M.); jgummert@hdz-nrw.de (J.G.); 6 Department of Cardiology, University Hospital Frankfurt, 60598 Frankfurt, Germany; zeiher@em.uni-frankfurt.de

**Keywords:** mitral valve surgery, sleep disordered breathing, mitral regurgitation

## Abstract

Background: Sleep disordered breathing (SDB) is a frequent comorbidity in cardiac disease patients. Nevertheless, the prevalence and relationship between SDB and severe primary mitral regurgitation (PMR) has not been well investigated to date. Methods: A cohort of 121 patients with significant PMR undergoing mitral valve surgery were prospectively enrolled and received a cardiorespiratory single night polygraphy screening using ApneaLink before surgery. Eighty-two of them underwent a follow-up examination including a follow-up single-night sleep study 3 months after surgery. Results: The mean age of patients was 65.3 ± 12.0 years. Sixty patients (49.6%) were female. The mean EuroSCORE II was 2.5 ± 2.4%. Initially, 91 (75.2%) patients presented with SDB, among whom 50.4% (46 patients, 38.0% of total cohort) were classified as moderate to severe. These patients tended to require significantly longer postoperative intensive care and mechanical ventilation. Among the 82 patients who completed follow-up exams, mitral valve surgery led to a significant reduction in relevant SDB (20.7%). The apnea-hypopnea index (from 11/h [4;18] to 4/h [3;14] (*p* = 0.04)), the oxygenation-desaturation index (from 8/h [3;18] to 5/h [3;12] (*p* = 0.008)) as well as the saturation time below 90% (from 32 min [13;86] to 18 min [5;36] (*p* = 0.005)), were all shown to be improved significantly. Conclusion: The prevalence of SDB is very high in patients with severe primary mitral regurgitation and may contribute to postoperative complications and prolonged intensive care. A significantly reduced but still high prevalence of SDB was observed 3 months after mitral valve surgery, highlighting the bidirectional relationship between SDB and heart failure.

## 1. Introduction

While sleep disordered breathing (SDB) is frequently underdiagnosed, it represents an independent risk factor for cardiac arrhythmia, progression of heart failure and increased mortality [1,2,3,4]. SDB is defined by episodes of apnea and hypopnea with accompanying hypoxemia and arousal from sleep resulting in disturbed sleep, recurrent sympathetic activation and impairment of cardiac function [5]. There are two main types of SDB: central (CSA) and obstructive sleep apnea (OSA). In this context, the duration of hypoxemic events, cycle length of apneic events and ventilation periods appear to depend on the cardiac function [6,7]. Longer-lasting respiratory events are generally more prevalent in patients with more impaired cardiac contractility, leading to pulmonary congestion and forward failure [6]. Furthermore, SDB may predispose patients to peri- and postoperative complications such as myocardial infarction, bleeding, prolonged hospital stay and respiratory complications in cardiac and also non-cardiac surgery [8,9]. Rupprecht et al. have demonstrated that severe SDB may increase the risk of death, septic and respiratory complications after elective coronary bypass grafting. In patients with severe aortic stenosis and pressure overload, SDB and CSA in particular are highly prevalent [10]. Additionally, increased left ventricular end-diastolic pressure following aortic regurgitation and volume overload in the pulmonary vasculature are known to favor and even worsen SDB [10,11], emphasizing the connection between SDB prevalence and affection of pulmonary vascular pressure by disordered cardiac flow-pattern.

Primary mitral regurgitation (PMR) due to degeneration of the valve leaflets is the most common aetiology for symptomatic severe mitral regurgitation, necessitating interventional or surgical therapy. Surgical mitral valve repair is the treatment of choice in cases of symptomatic severe PMR [12]. Mitral regurgitation leads to a volume overload in the left atrium, and consequently to continuously elevated pressure in the pulmonary vascular bed [11].

However, since pathologies of the aortic valve which affect the pulmonary vascular bed show a connection to SDB [10], mitral regurgitation—a direct dysfunction of the separation of the low-pressure system of the pulmonary veins and the left atrium from the left ventricular high-pressure system—is likely to have an impact on SDB. As only limited knowledge with regard to this is available, the current manuscript analyzes the prevalence, and changes in SDB in patients with PMR undergoing mitral valve surgery.

## 2. Methods

Patients with PMR, admitted to the Herz- und Diabeteszentrum NRW for treatment evaluation between July 2015 and May 2017, were prospectively enrolled. The decision for surgical treatment was made after a heart team discussion in each case individually.

The main exclusion criteria were previously diagnosed SDB, a relevant lung disease including precapillary pulmonary hypertension, congenital heart disease, previous cardiac surgery and the need for additional cardiac surgical procedures.

Eligible patients for surgery underwent sleep analysis before and 3 months after surgery. In addition, New York Heart Association (NYHA) classification, electrocardiography, echocardiographic examinations and the 6 min walking test to assess functional exercise capacity were performed. The study was approved by the local Ethics Committee of the Ruhr University Bochum (No 28/2014) and carried out in accordance with the Declaration of Helsinki and was registered at www.clinicaltrials.gov in 20 November 2014 (NCT02296710).

### 2.1. Echocardiography

All participants underwent standard transthoracic echocardiography (EPIQ 7, Philips Electronics, Eindhoven, The Netherlands). The images were stored digitally in a rapid mass storage system. Echocardiographic examinations were performed by highly qualified medical staff and were analyzed by the same echocardiographer with long-term experience. The analyses, including grading of the mitral regurgitation, were performed following the recommendations of the American and European Societies of Echocardiography [13,14]. LV ejection fraction was assessed using the Simpson’s method from apical two- and four-chamber views. In cases with irregular cardiac rhythm (e.g., atrial fibrillation, frequent atrial or ventricular ectopy) at least 5 loops were recorded, and the average values have been provided.

### 2.2. Sleep Disordered Breathing

Polygraphic single night examination was done as previously described [15]. The sleep analysis (ApneaLink™ Air and ApneaLink Reporting System Version 10.20, Resmed Healthcare, Martinsried, Germany) without a chest strap to assess the chest wall motion was performed as a single sleep study 2 to 7 days before and 3 months after surgery, between 10:00 p.m. and 6:00 a.m., in accordance with the recommendations of the American Academy of Sleep Medicine [16]. Apnea was defined as a ≥90% drop in the flow signal compared to the pre-event baseline for a period of at least 10 s [16]. Hypopnea was defined as a flow-drop of 30% below the pre-event baseline for a period of at least 10 s and an oxygen desaturation of at least 3% compared with the pre-event baseline. The apnea hypopnea index (AHI) was calculated as the number of episodes of apnea and hypopnea per hour of estimated sleep. The oxygen desaturation index was calculated as the number of oxygen desaturations per hour of sleep. Mild, moderate and severe SDB were defined as an AHI between 5 and 14 events per hour, 15 to 29 events per hour and ≥30 events per hour, respectively [16].

### 2.3. Statistical Analysis

Statistical analysis was performed using the SPSS-Software (Version 21, IBM, New York, NY, USA). Results are given as mean values ± standard deviation or median with interquartile range for continuous variables, or as percentages for categorical variables, unless otherwise specified. Baseline data were checked for normal distribution with the Shapiro–Wilk test. In the absence of normal distribution, the Mann–Whitney U test was used. Calculation of the pre- and postoperative differences was performed by two-tailed paired *t* test or Wilcoxon signed rank test depending on normal distribution. A value of *p* < 0.05 was considered statistically significant.

## 3. Results

**Figure 1:**
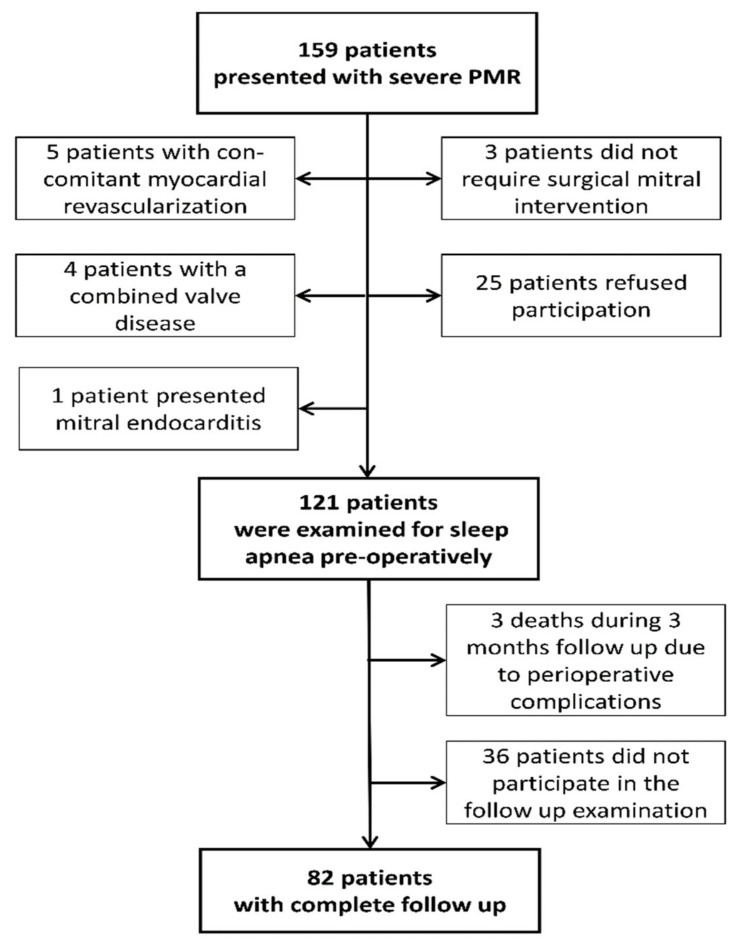
Patient Enrollment and Follow-up. PMR: primary mitral regurgitation.

A total of 159 patients with PMR were admitted and evaluated for mitral valve surgery. Five of them required myocardial revascularization and five patients presented with a combined valve disease which had to be addressed. In three cases, the heart team voted against surgical treatment, 25 patients refused participation in the study and one patient was admitted for mitral valve endocarditis. Finally, 121 patients underwent sleep apnea testing before surgery. A second polygraphic examination was planned for 3 months after surgery. Three patients died from complications after surgery before the follow-up and 36 patients failed to complete the follow-up controls (refusal by patient or technical difficulties). Eventually, 82 patients completed all examinations (Figure 1).

Eighty-five patients (70.2%) underwent mitral valve repair, while valve replacement was performed on 36 patients (29.8%).

Demographic data of the patients are compiled in Table 1. The mean age of the patients was 65.3 ± 12.0. Sixty patients (49.6%) were female. Patients presented with a mean EuroSCORE II of 2.5 ± 2.4% (low to intermediate perioperative risk) [17]. Left ventricular ejection fraction was normal in the presence of severe mitral regurgitation with a mean effective regurgitant orifice area of 44 ± 5 mm^2^, a mean regurgitant volume of 68 ± 3 mL and a mean vena contracta of 7.5 ± 0.6 mm.

Three months after surgery, a significant reduction in the ejection fraction was recorded along with a significantly reduced left ventricular end-diastolic diameter and volume. The left atrial volume was also significantly reduced. Detailed echocardiographic parameters before and after surgery are listed in Table 2.

Heart failure symptoms significantly improved after surgery, with a reduction in the NYHA class (at baseline 66.3% were classified as NYHA class III or IV, 3 months after surgery 85.2% were classified as NYHA class I or II; *p* < 0.001;) and an increased walking distance in the 6-min walking test (from 372.3 ± 31.6 m to 424.8 ± 117.0 m (*p* < 0.001)). Before surgery, 36.8 % of the patients presented with atrial fibrillation. This percentage decreased significantly to 22.6% three months after surgery (*p* = 0.03).

**Figure 2:**
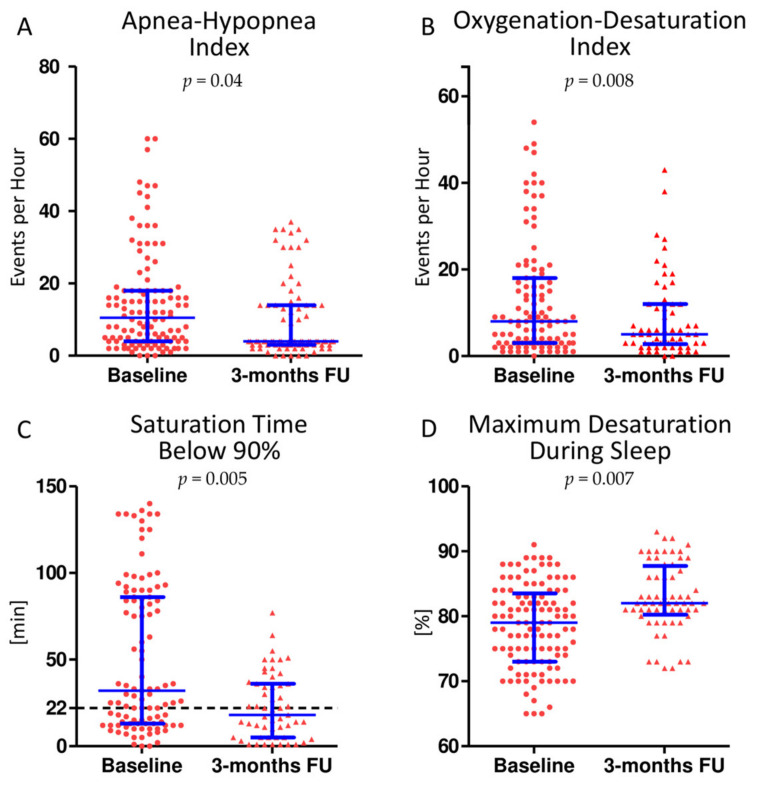
Sleep disordered breathing (SDB) parameters improve after elimination of mitral regurgitation. Apnea hypopnea index (**A**), the oxygenation desaturation index (**B**) and the oxygen saturation time below 90% (**C**) improved significantly three months after mitral valve surgery. In addition, the oxygen saturation during sleep did not decrease as much as before surgery (**D**). The prognostically relevant time of oxygen saturation of 22 min (black line, (**C**)) was no longer exceeded in most patients after surgery. Scatter dot plots are lined at mean with interquartile range.

At baseline, 91 out of 121 patients (75%) suffered from a clinically significant sleep breathing disorder. Among these, 50.4% (46 patients, 38% of the total) were of moderate to severe degree (AHI ≥ 15/h). At follow-up, 30 out of 82 patients (36.6%) presented with an ongoing SDB. Seventeen cases (20.7%) were classified as moderate to severe. AHI decreased significantly from 11/h [4;18] to 4/h [3;14] (*p* = 0.04). The oxygenation desaturation index showed a significant reduction from 8/h [3;18] to 5/h [3;12] (*p* = 0.008). The time of saturation below 90% (T_<90%_) was significantly shorter after surgery (from 32 min [13;86] to 18 min [5;36]; *p* = 0.005), with significantly fewer maximum desaturations (from 78 ± 8% to 82 ± 7% *p* = 0.007). The changes in sleep apnea parameters associated with mitral valve surgery are illustrated in Figure 2. Among patients with relevant SDB at baseline (AHI ≥ 15/h), SDB parameters improved to a greater extent (AHI from 22/h [16;40] to 14/h [5;30] (*p* < 0.001), ODI from 19/h [6;36] to 6/h [3;17] (*p* = 0.0063), T_<90%_ from 75 min [98;24] to 23 min [12;40] (*p* < 0.001) and the maximum desaturation during sleep from 77% [73;81] to 83% [79;83] (*p* = 0.047). No patient received specific SDB treatment before the second polygraphy. Patient weight did not significantly change (from 72.4 ± 5.3 kg to 73.1 ± 5.9 kg (*p* = 0.7)) between examinations.

New onset of atrial fibrillation was observed in four patients (three patients with AHI ≥ 15/h and one patient with AHI < 15/h). The rate of major adverse cardiac and cerebrovascular events (MACCE) showed no significant difference in patients with and without relevant SDB (AHI ≥ 15/h: 4.3%; AHI < 15/h: 2.7% (*p* = 0.52)). However, in patients with moderate to severe SDB, a significantly longer need for intensive care (AHI < 15/h: 22 h [19;61.75]; AHI ≥ 15/h: 37.5 h [21.75; 124.75] *p* = 0.01) and a longer ventilation time (AHI < 15/h: 7.83 h [6.12; 10.25]; AHI ≥ 15/h: 8.9 h [6.69;19.56] *p* = 0.02) was observed. The total duration of hospitalization did not differ significantly from patients with an AHI ≤ 15/h (AHI < 15/h: median 13 [11;14]; AHI ≥ 15/h: median 13 [11;16] *p* = 0.113).

## 4. Discussion

**Figure 3:**
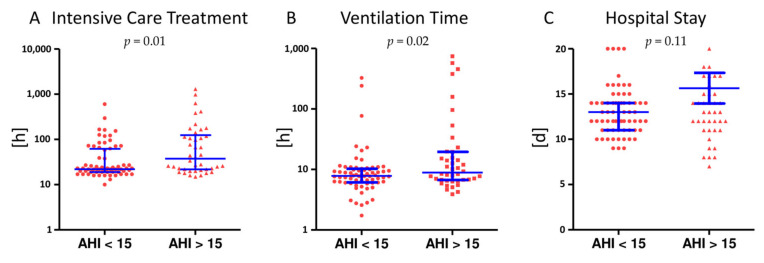
Impact of SDB on perioperative outcome.

This study represents the first prospective assessment of SDB in the setting of mitral valve surgery, revealing a very high prevalence of undiagnosed SDB (75%) in patients with symptomatic severe PMR. In addition, moderate to severe SDB (AHI ≥ 15/h) has an adverse effect on the perioperative outcome with a prolonged ventilation time and longer need for intensive care (Figure 3). In a mid-term follow-up after 3 months the rate of SDB still remained high, although SDB may significantly improve, as indicated by a much shorter T_<90%_ time, in particular in patients with relevant SDB at baseline (AHI ≥ 15/h).

The need for intensive care and mechanical ventilation time was significantly longer in patients with moderate to severe SDB (AHI ≥ 15/h) (**A**, **B**) while the total duration of hospital stay did not differ significantly (**C**). Scatter dot plots are lined at the mean with interquartile range.

### 4.1. Prevalence and Dynamic of Undiagnosed SDB

While several studies alleged an association between congestive heart failure and SDB or severe aortic stenosis and SDB, there is little information available on the prevalence of SDB in patients with severe PMR [10,18,19,20]. In our study, the total prevalence of SDB was 75%, while undiagnosed SDB with an AHI ≥ 15/h was detected in 38% of the patients with symptomatic PMR. This number dropped 3 months later to 20%, but remained very high. Spiesshöfer et al. demonstrated a correlation between SDB and severe secondary mitral regurgitation in a small study population of twenty patients with high operative risk who underwent percutaneous edge-to-edge mitral valve repair [11]. The mean age in these patients was 80 ± 6 years and the mitral regurgitation was mostly of functional/secondary origin. Nevertheless, the age dependency of SDB remains unclear because of the high prevalence of SDB in the population of elderly people [21]. Our study population is significantly younger and comprises mitral regurgitation of primary/degenerative origin, yet there is a very high prevalence of SDB (75%) which is probably explained by the interrelation between cardiac filling pressures and chronic heart failure on one side and SDB on the other [22]. A T_<90%_ of more than 22 minutes appears to be a prognostically relevant parameter for nocturnal hypoxemic stress and is associated with higher all-cause mortality [1]. In this study the T_<90%_ time, in addition to AHI, improved remarkably after surgery, which clearly shows an improved SDB burden. After mitral valve surgery, the LA-volume index generally decreased, hence reducing the volume overload and pressure in the pulmonary vascular bed and thereby improving SDB severity [23]. The myocardial performance is optimized after eliminating the regurgitant fraction and producing an antegrade stroke volume only (or mainly). Due to the economization of heart function, heart failure symptoms such as dyspnea (NYHA class) and exercise capacity (6 min-walked distance), but also SDB, improved significantly, emphasizing the particular association between SDB and heart failure [22]. Besides this, atrial fibrillation—a common comorbidity in mitral regurgitation due to left atrial dilation [24]—decreased along with SDB. After elimination of the regurgitant fraction, the mentioned pulmonary congestion decreases, which may also lead to a reduction in atrial fibrillation [24]. In addition, the interdependency of atrial fibrillation and SDB could also contribute to an improved atrial fibrillation burden due to the attenuated SDB severity [25].

### 4.2. Impact of SDB on the Postoperative Outcome

Although the total hospital stay did not differ, moderate to severe SDB had an adverse effect on the postoperative need for intensive care and the ventilation time in our study. Moderate to severe SDB in the context of coronary artery bypass grafting is known to worsen the postoperative outcome, with prolonged hospital stay and a greater risk of postoperative hemodynamic instability [8]. In general, newer postoperative care protocols aim for a fast-track recovery, including early extubation and mobilization to avoid postoperative pneumonia and complications contributing to reduced intensive care needs and in-hospital costs [26]. Based on this concept, detection of SDB before surgery could help to optimize preoperative conditions and prevent an adverse postoperative outcome [10]. As is well understood, the duration of mechanical ventilation correlates closely with ventilator-associated complications [27], thus, any postoperative care protocol aims to minimize ventilation time after surgery. In this regard, SDB resembles a predisposing comorbidity to prolong ventilation time and, accordingly, the need for intensive care. Against this background, in order to minimize the mentioned risks, detection of SDB before surgery will be of crucial interest for medical research and practice.

### 4.3. Limitations

This study’s validity is limited by technical issues, as it was conducted with the Apnealink portable polygraphic system without a chest strap to assess chest wall motion, which does not allow for differentiation between central and obstructive sleep apnea. This, however, would be of tremendous interest. Moreover, polysomnographic examination is the gold standard for diagnosing SDB enabling precise distinction between OSA and CSA and evaluation of sleep quality, e.g., by analyzing sleep stages. 

## 5. Conclusions and Clinical Implication

In patients with symptomatic PMR, the presence of SDB should be assessed prior to surgery and clinicians should begin with a non-invasive ventilation therapy and be prepared for a longer need for intensive care. However, our data demonstrate that the economization of cardiac function with higher contractile effectiveness after mitral valve surgery may result in an improvement in SDB, highlighting the bidirectional relation between SDB and heart failure. Nevertheless, a remarkably high SDB rate (20% with AHI ≥ 15/h) with need of follow-up and therapy remains. Therefore, in the context of a complementary strategy for the management of heart failure, the detection and treatment of SDB is essential.

## Figures and Tables

**Table 1:** Baseline Characteristics of the Collective.

Baseline Characteristics		
Age		65.3 ± 12.0
Female		47.8% (60)
Systolic Blood pressure		118.4 ± 19.3 mmHg
Diastolic Blood pressure		81.2 ± 11.5 mmHg
Coronary Artery Disease		10.9% (13)
Body Mass Index [kg/m^2^]		25.9 ± 5.1
EuroSCORE I		8.2 ± 7.3%
EuroSCORE II		2.5 ± 2.4%
Peripheral Artery Disease		3.3% (3)
Stroke		11.6% (14)
Diabetes Mellitus		9.1% (11)
Renal Insufficiency (GFR categories ≥ G3b)		9.9% (12)
Chronic Obstructive Pulmonary Disease		9.1% (11)
Left Bundle Branch Block		3.3% (4)
Previous Myocardial Infarction		3.3% (4)
Prior Percutaneous Coronary Intervention		5.1% (7)
Prior Cardiac Surgery		11.6% (14)
Atrial Fibrillation		34.7% (42)
Permanent Pacemaker		4.1% (5)
**Medications**	B-Blocker	58.7% (71)
	Diuretics	41.3% (50)
	Other Drugs (including ACE/AT1 inhibitors and calcium antagonists)	47.1% (57)

**Table 2:** Morphological and functional changes in the left ventricle 3 months after mitral valve surgery.

Echocardiographic Parameters	Before Surgery	After Surgery	*p*-Value
MR PISA radius adjusted to Nyquist-limit 30–40 cm/s [mm]	11 ± 1	Ø	
MR effective regurgitant orifice area [mm^2^]	44 ± 5	Ø	
MR regurgitant volume [mL]	68 ± 3	Ø	
MR vena contracta [mm]	7.5 ± 0.6	Ø	
LA volume [mL]	140 ± 14	95 ± 81	<0.001
LA volume index [mL/m^2^]	74 ± 15	48 ± 35	<0.001
LV enddiastolic diameter [mm]	57 ± 9	54 ± 9	<0.001
LV endsystolic diameter [mm]	40 ± 2	41 ± 5	0.6
LV enddiastolic volume [mL]	160 ± 10	136 ± 11	<0.001
LV endsystolic volume [mL]	70 ± 41	69 ± 90	0.6
LV ejection fraction Simpson [%]	56 ± 20	49 ± 15	<0.001
Total Stroke volume [mL]	91 ± 63	67 ± 35	<0.001

Term explanation: LA: Left atrial; LV: Left ventricular; MR: Mitral regurgitation; PISA: Proximal isovelocity surface area.

## Data Availability

The data presented in this study are available on request from the corresponding author. The data are not publicly available due to privacy and ethical restrictions.
